# Visual impairment and blindness in the Xingu Indigenous Park – Brazil

**DOI:** 10.1186/s12939-021-01536-w

**Published:** 2021-08-30

**Authors:** Arthur Gustavo Fernandes, Monica Alves, Roberta Andrade e Nascimento, Natalia Yumi Valdrighi, Rafael Cunha de Almeida, Celso Takashi Nakano

**Affiliations:** 1 Associação Médicos da Floresta, Sao Paulo, Brazil; 2 Department of Ophthalmology and Visual Sciences, Federal University of Sao Paulo - Paulista Medical School, Sao Paulo, Brazil; 3 Department of Ophthalmology and Otorhinolaryngology, University of Campinas, Campinas, Brazil; 4 Department of Ophthalmology, ABC Medical School, Santo Andre, Brazil; 5 Department of Ophthalmology, University of Sao Paulo, Sao Paulo, Brazil

**Keywords:** Blindness, Indigenous, Ophthalmology, Public Health, Visual Impairment

## Abstract

**Background:**

Most estimates of visual impairment and blindness worldwide do not include data from specific minority groups as indigenous populations. We aimed to evaluate frequencies and causes of visual impairment and blindness in a large population sample from the Xingu Indigenous Park.

**Methods:**

Cross-sectional study performed at Xingu Indigenous Park, Brazil, from 2016 to 2017. Residents from 16 selected villages were invited to participate and underwent a detailed ocular examination, including uncorrected (UVA) and best-corrected visual acuity (BCVA). The main cause of UVA < 20/32 per eye was determined.

**Results:**

A total of 2,099 individuals were evaluated. Overall, the frequency of visual impairment and blindness was 10.00% (95% CI: 8.72–11.29%) when considering UVA, decreasing to 7.15% (95% CI: 6.04–8.25%) when considering BCVA. For each increasing year on age, the risk  of being in the visually impaired or blind category increased by 9% (*p* < 0.001). Cataracts (39.1%) and uncorrected refractive errors (29.1%) were the most frequent causes of visual impairment and blindness in this population. The main causes among those aged 45 years and more were cataracts (54.5%) while refractive errors were the main cause in adults aged 18 to 45 years (50.0%) and children up to 18 years old (37.1%).

**Conclusions:**

A higher frequency of visual impairment and blindness was observed in the indigenous population when compared to worldwide estimates with most of the causes being preventable and/or treatable. Blindness prevention programs should focus on accessibility to eye exam, cataract surgeries and eyeglass distribution.

## Background

According to the most recent estimates, there are 43.3 million people blind worldwide representing a prevalence of 5.25 cases per 1,000 persons (95% CI: 4.58 – 5.87) and 295.3 million people moderate to severe visually impaired representing a prevalence of 35.8 cases per 1,000 persons (95% CI: 32.4 – 39.2) [1]. The main causes for blindness are reported as cataract, glaucoma, uncorrected refractive errors, age-related macular degeneration and diabetic retinopathy, while the main causes for visual impairment are uncorrected refractive errors, cataract, age-related macular degeneration, glaucoma and diabetic retinopathy [2].

Most estimates, however, do not include data from specific minority populations which are expected to present higher frequencies of visual impairment [3–5]. As a result, the burden of visual impairment and blindness may be underestimated and the public health policies derived from it may insufficiently attend the demand of those minority groups. Those groups are often under-represented in research because population-based studies aim to select samples that represent the overall population and/or due to the low response rate from those specific groups even when they are included in the sampling [6, 7].

Indigenous populations are a minority ethnic group and are considered one of the most marginalized and disadvantaged people worldwide [8]. A recent systematic review on visual loss among indigenous populations has shown a lack of data on the burden of visual loss in most countries and has pointed the importance of improvements in quality and number of researches about eye health and eye care in indigenous communities [9].

Brazilian indigenous groups represent approximately 500 thousand individuals, counting for near 1% of the Brazilian population [10]. The epidemiological profile of these individuals, however, is still little known mainly due to the scarcity of population-based studies involving indigenous groups and the absence of an efficient integrated system for reporting data. The few data available reveal high incidence of acute respiratory and gastrointestinal infections, malaria, tuberculosis, sexually transmitted diseases and malnutrition, with great variability among different communities. There is also an increase in cases of hypertension, diabetes, alcoholism, depression and suicide, related to changes in the lifestyle in the recent years [11, 12].

Studies related to the eye health of indigenous groups are even more scarce, with very few data reported from populations resident in the North Region of the country. The latest published data referring to the eye health of the inhabitants of the Xingu Indigenous Park are dated from 1996 investigating frequency of trachoma and from 2003 analyzing refractive errors [13, 14].

The Xingu Indigenous Park is located in the north of Mato Grosso state in the Brazilian Midwest region and it is the largest indigenous reserve in the country with 2.6 million hectares of area where approximately 6,000 inhabitants of 16 different ethnicities live distributed in 58 villages.

The project named “Olhos do Xingu” is an initiative of the non-profitable organization Associação Médicos da Floresta. This Brazilian non-profit civil entity was founded in 2016 by doctors and managers with experience in voluntary provision of health services in indigenous communities located in areas of difficult access. The Project aims to address the demand for specialized ophthalmological assistance to the indigenous communities by providing complete ocular exam, prescription and donation of glasses, besides cataract and pterygium surgeries and other specific treatments when needed.

The purpose of the current study is to evaluate the frequencies and causes of visual impairment and blindness in a large population sample from the Xingu Indigenous Park.

## Methods

### Study population

The Olhos do Xingu Project visited 16 villages between 2016 and 2017. All individuals residing in those villages were invited for a complete eye examination, regardless of age, sex or visual complaint. The 16 villages were selected from the 58 enumerated using the following criteria: most populated villages, feasible access through the Xingu river branches, and agreement to participation from the local leaders. Before planning the expedition and data collection, a professional from the Associação Médicos da Floresta went to each site to explain the project to local leaders and for the written informed consent acquisition. This study was approved by The Brazilian National Research Ethics Committee and was carried out in accordance with the tenets of the Declaration of Helsinki.

Teams of ophthalmologists, ophthalmic technologists, clinical doctors and logistics professionals travelled to the Xingu Indigenous Park carrying ophthalmological equipment and supplies in order to set up itinerant ophthalmic clinics in each village included in the project.

On the data collection day, a member of the team was responsible to visit each of the residences in the village in order to reinforce the invitation to participate and to explain the importance of the eye examination for both children and adults aiming to increase the project coverage.

### Ophthalmic exam

Demographic data, such as name, identification number, age, and ethnicity were collected before the eye exam.

Distance visual acuity (VA) was measured without correction (UVA) and subsequently with the best correction indicated in the subjective refraction test (BCVA). The ophthalmologist measured the visual acuity using a Snellen chart with “E” optotypes positioned at a distance of 6 m. Visual acuity was measured in each eye separately up to the line with the smallest optotypes read correctly and this value was recorded using the Snellen fraction at 20 feet.

The visual status of each eye based on the results of visual acuity was classified as: No visual impairment when VA ≥ 20/32; Mild visual impairment when VA < 20/32 to VA ≥ 20/63; Moderate visual impairment when VA < 20/63 to VA ≥ 20/200; Severe visual impairment when VA < 20/200 to VA ≥ 20/400; and Blindness when VA < 20/400 [1].

After visual acuity test, automated and subjective refractions were performed, and the best-corrected visual acuity was determined by the ophthalmologist. A comprehensive slit lamp examination detailed eyelids, cornea, lens and conjunctiva. Any abnormalities in the eye anterior segment were noted and specified. Posterior segment was evaluated under pupil dilation.

Main causes of visual impairment/blindness were diagnosed and recorded by the ophthalmologist using the following options: Not visually impaired (UVA  ≥ 20/32); Refractive error (UVA < 20/32 and BCVA ≥ 20/32); Amblyopia; Cataract; Posterior capsule opacity; Corneal opacity / scar; Absent globe; Glaucoma; Age-related macular degeneration; Diabetic retinopathy; Retinal detachment; Other retinal / choroidal abnormalities; Pterygium; Other cause.

Proper explanation was provided for each participant after the exam in order to guarantee a best decision-making treatment. When prescribed, glasses were provided free of charge on site for simple spherical corrections or delivered up to one month after the exams as they were manufactured in a different state. Cataract or pterygium surgeries were scheduled for the subsequent expedition organized by the Associação Médicos da Floresta. Other specific treatment, such as glaucoma or strabismus were referred through the Brazilian national insurance system.

### Statistical analysis

Statistical analyses were performed using Stata/SE Statistical Software, Release 14.0, 2015 (Stata Corp, College Station, Texas, USA). Frequency tables were used for descriptive analysis. Disease frequencies were calculated from the number of cases in the study population along with the 95% confidence intervals considering the visual acuity from the better-seeing eye. The associations between variables were evaluated through Poisson regressions. *P* values ≤ 0.05 were considered statistically significant.

Causes were evaluated in both person and eye level. To report a principal cause of visual impairment (UVA worse than 20/32 in the better-seeing eye) at the person level, the main cause was determined considering same cause for both eyes or different causes in the two eyes, specifying the combinations [15, 16]. To report the principal cause at the eye level, each eye was evaluated independently considering its visual status regardless of the contralateral eye.

## Results

According to census data from the Brazilian Special Indigenous Sanitary District (DSEI), the total resident population of the 16 selected villages comprises 3,674 individuals. Along the study period, 2,099 individuals were evaluated representing an overall coverage of 57.13%. Table 1 indicated the total number of enumerated and examined participants, according to the age group.

**Table 1:** Enumerated and examined participants

Age category	Enumerated (n)	Examined (n)	Coverage (%)
0 to 17 years old	2105	827	39.29%
18 to 44 years old	1080	816	75.56%
45 years or more	489	456	93.25%
Total	3674	2099	57.13%

Although majority, when considering the absolute number of examined individuals, those under 18 years old showed the lowest coverage in the population while the older participants showed a higher adherence into the project with coverages above 75% (< 0.0001).

Table 2 shows the visual status of the examined participants considering the visual acuity from the best-seeing eye.

**Table 2:** Visual status considering the visual acuity from the best-seeing eye

	**No visual impairment *(VA* ≥ *20/32)***	**Mild visual impairment** ***(VA* < *20/32 to VA* ≥ *20/63)***	**Moderate visual impairment *(VA* < *20/63 to VA* ≥ *20/200)***	**Severe visual impairment *(VA* < *20/200 to VA* ≥ *20/400)***	**Blindness *(VA* < *20/400)***	**Total**
	*N (%)*	*N (%)*	*N (%)*	*N (%)*	*N (%)*	*N (%)*
**0 to 17 years old**	818 (98.91) 824 (99.64)	4 (0.48) 2 (0.24)	3 (0.36) 0 (0.00)	0 (0.00) 0 (0.00)	2 (0.24) 1 (0.03)	827 (100.00)
**18 to 44 years old**	776 (95.10) 799 (97.92)	24 (2.94) 9 (1.10)	10 (1.23) 6 (0.74)	0 (0.00) 0 (0.00)	6 (0.74) 2 (0.25)	816 (100.00)
**45 years or more**	295 (64.69) 326 (71.49)	66 (14.47) 51 (11.18)	64 (14.04) 50 (10.96)	2 (0.44) 2 (0.44)	29 (6.36) 27 (5.92)	456 (100.00)
**Total**	1889 (90.00) 1949 (92.85)	94 (4.48) 62 (2.95)	77 (3.67) 56 (2.67)	2 (0.10) 2 (0.10)	37 (1.76) 30 (1.43)	2099 (100.0)

First line representing uncorrected visual acuity (UVA) and second line representing best-corrected visual acuity (BCVA). *VA* Visual acuity

**Fig. 1:**
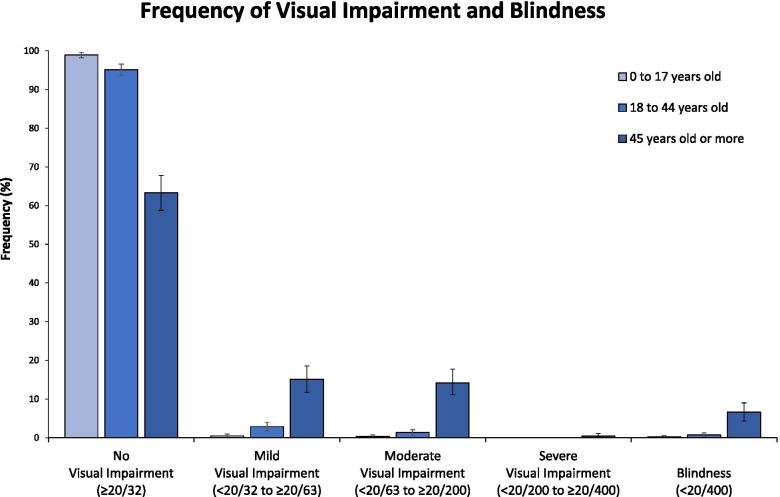
Frequency of visual impairment and blindness according to age groups

Overall, we observed a frequency of visual impairment and blindness of 10.00% (95% CI: 8.72 – 11.29%) when considering the UVA, decreasing to 7.15% (95% CI: 6.04 – 8.25%) when considering BCVA. Figure 1 represents the frequencies of visual impairment and blindness in the population considering the UVA in the different age groups.

Poisson regression for visual impairment and blindness adjusted for age and village of residence showed that age is associated with visual impairment and blindness so that for each increasing year on age the risk of being in the visually impaired or blind category increases by 9% (*p* < 0.001).

After completing the eye exam protocol, the cause of visual impairment was stated among the options described in the methodology section. Table 3 shows the principal cause of bilateral moderate to severe visual impairment (MSVI) (VA worse than 20/200 to 20/400 or better) and blindness (VA worse than 20/400) on a per-person basis.

**Table 3:** Principal cause of moderate to severe visual impairment (UVA worse than 20/63 to 20/400 or better) and blindness (UVA worse than 20/400) per person considering same cause in both eyes and combinations of distinct causes for each eye

Principal cause	Moderate to Severe Visual Impairment			Blindness			Total		
	0 to 17	18 to 44	45 and more	0 to 17	18 to 44	45 and more	0 to 17	18 to 44	45 and more
	*N (%)*	*N (%)*	*N (%)*	*N (%)*	*N (%)*	*N (%)*	*N (%)*	*N (%)*	*N (%)*
***Same cause in both eyes***	***3 (100.00)***	***10 (100.00)***	***54 (81.82)***	***2 (100.00)***	***6 (100.00)***	***18 (62.07)***	***5 (100.00)***	***16 (100.00)***	***72 (75.79)***
Cataract			34 (51.51)			14 (48.28)			48 (50.53)
Uncorrected refractive error^a^	2 (66.33)	3 (30.00)	10 (15.15)	1 (50.00)	2 (33.33)		3 (60.00)	5 (31.25)	10 (10.53)
Retinal abnormalities^b^		2 (20.00)	3 (4.54)		1 (16.66)			3 (18.75)	3 (3.16)
Amblyopia	1 (33.33)	1 (10.00)			2 (33.33)		1 (20.00)	3 (18.75)	
Corneal opacity / scar		2 (20.00)				1 (3.45)		2 (12.50)	1 (1.05)
Pterygium			3 (4.54)						3 (3.16)
Glaucoma						1 (3.45)			1 (1.05)
Other causes		2 (20.00)	4 (6.06)	1 (50.00)	1 (16.66)	2 (6.90)	1 (20.00)	3 (18.75)	6 (6.32)
***Distinct causes for each eye***			***12 (18.18)***			***11 (37.93)***			***23 (24.21)***
Cataract | Retinal abnormalities^b^			4 (6.06)			3 (10.34)			7 (7.37)
Cataract | Corneal opacity / scar			3 (4.54)			2 (6.90)			5 (5.26)
Cataract | Posterior capsule opacity						2 (6.90)			2 (2.10)
Cataract | Absent globe						1 (3.45)			1 (1.05)
Cataract | Other causes			4 (6.06)			2 (6.90)			6 (6.32)
Uncorrected refractive error^a^ | Retinal abnormalities^b^			1 (1.51)						1 (1.05)
Retinal abnormalities | Corneal opacity / scar						1 (3.45)			1 (1.05)
**Total**	**3 (100.00)**	**10 (100.00)**	**66 (100.00)**	**2 (100.00)**	**6 (100.00)**	**29 (100.00)**	**5 (100.00)**	**16 (100.00)**	**95 (100.00)**

^a^ including only eyes that improved to VA ≥ 20/32 after subjective refraction

^b^ Retinal abnormalities such as: age-related macular degeneration, diabetic retinopathy, retinal detachment, macular hole, and chorioretinitis

Bilateral uncorrected refractive error was the main cause of both moderate to severe visual impairment (MSVI) and blindness in participants aged 0 to 17 years old (MSVI: 66.3%, Blind: 50.0%) and in participants aged 18 to 44 years old (MSVI: 60.0%, Blind: 33.3%) while bilateral cataract was the main cause in participants 45 years and older (MSVI: 51.5%, Blind: 48.3%).

Out of the total 4,198 evaluated eyes, 3,576 (85.2%) had no visual impairment (UVA ≥ 20/32). Table 4 describes the main cause of visual acuity < 20/32 in the remaining eyes (N = 622) by age groups, independently, regardless of the individual visual status.

**Table 4:** Principal cause of visual impairment and blindness by eye

	**Mild visual impairment**			**Moderate visual impairment**			**Severe visual impairment**			**Blindness**			***Any impairment level***			**OVERALL**
	0 to 17	18 to 44	45 and more	0 to 17	18 to 44	45 and more	0 to 17	18 to 44	45 and more	0 to 17	18 to 44	45 and more	**0 to 17**	**18 to 44**	**45 and more**	
	*N(%)*	*N(%)*	*N(%)*	*N(%)*	*N(%)*	*N(%)*	*N(%)*	*N(%)*	*N(%)*	*N(%)*	*N(%)*	*N(%)*	*N(%)*	*N(%)*	*N(%)*	*N(%)*
**Cataract**	1 (4.35)	3 (3.16)	145 (55.77)	0 (0.00)	1 (2.63)	32 (40.00)	0 (0.00)	0 (0.00)	5 (100.00)	0 (0.00)	0 (0.00)	56 (60.87)	***1*** ***(2.86)***	***4*** ***(2.67)***	***238*** ***(54.46)***	**243** **(39.07)**
**Uncorrected refractive error**^**a**^	10 (43.48)	54 (56.84)	65 (25.00)	1 (16.67)	17 (44.74)	26 (32.50)	0 (0.00)	0 (0.00)	0 (0.00)	2 (33.33)	4 (23.53)	2 (2.17)	***13*** ***(37.14)***	***75*** ***(50.00)***	***93*** ***(21.28)***	**181** **(29.10)**
**Retinal abnormalities**^**b**^	0 (0.00)	6 (6.32)	8 (3.08)	0 (0.00)	3 (7.89)	3 (3.75)	0 (0.00)	0 (0.00)	0 (0.00)	0 (0.00)	4 (23.53)	10 (10.87)	***0*** ***(0.00)***	***13*** ***(8.67)***	***21*** ***(4.81)***	**34** **(5.47)**
**Amblyopia**	6 (26.09)	6 (6.32)	1 (0.38)	3 (50.00)	3 (7.89)	2 (2.50)	0 (0.00)	0 (0.00)	0 (0.00)	1 (16.67)	7 (41.18)	0 (0.00)	***10*** ***(28.57)***	***16*** ***(10.67)***	***3*** ***(0.69)***	**29** **(4.66)**
**Corneal opacity / scar**	2 (8.70)	4 (4.21)	1 (0.38)	2 (33.33)	5 (13.16)	2 (2.50)	0 (0.00)	0 (0.00)	0 (0.00)	0 (0.00)	0 (0.00)	6 (6.52)	***4*** ***(11.43)***	***9*** ***(6.00)***	***9*** ***(2.06)***	**22** **(3.54)**
**Pterygium**	0 (0.00)	3 (3.16)	9 (3.46)	0 (0.00)	1 (2.63)	5 (6.25)	0 (0.00)	0 (0.00)	0 (0.00)	0 (0.00)	0 (0.00)	1 (1.09)	***0*** ***(0.00)***	***4*** ***(2.67)***	***15*** ***(3.43)***	**19** **(3.05)**
**Posterior capsule opacity**	0 (0.00)	2 (2.11)	6 (2.31)	0 (0.00)	0 (0.00)	0 (0.00)	0 (0.00)	0 (0.00)	0 (0.00)	0 (0.00)	0 (0.00)	3 (3.26)	***0*** ***(0.00)***	***2*** ***(1.33)***	***9*** ***(2.06)***	**11** **(1.77)**
**Glaucoma**	0 (0.00)	0 (0.00)	0 (0.00)	0 (0.00)	0 (0.00)	0 (0.00)	0 (0.00)	0 (0.00)	0 (0.00)	0 (0.00)	0 (0.00)	4 (4.35)	***0*** ***(0.00)***	***0*** ***(0.00)***	***4*** ***(0.92)***	**4** **(0.64)**
**Absent globe**	0 (0.00)	0 (0.00)	0 (0.00)	0 (0.00)	0 (0.00)	0 (0.00)	0 (0.00)	0 (0.00)	0 (0.00)	0 (0.00)	0 (0.00)	1 (1.09)	***0*** ***(0.00)***	***0*** ***(0.00)***	***1*** ***(0.23)***	**1** **(0.16)**
**Other causes**	4 (17.39)	17 (17.89)	25 (9.62)	0 (0.00)	8 (12.50)	10 (12.50)	0 (0.00)	0 (0.00)	0 (0.00)	3 (50.00)	2 (11.76)	9 (9.78)	***7*** ***(20.00)***	***27*** ***(10.07)***	***44*** ***(10.07)***	**78** **(12.54)**
**Total**	23 (100.00)	95 (100.00)	260 (100.00)	6 (100.00)	38 (100.00)	80 (100.00)	0 (0.00)	0 (0.00)	5 (100.00)	6 (100.00)	17 (100.00)	92 (100.00)	***35*** ***(100.00)***	***150*** ***(100.00)***	***437*** ***(100.00)***	**622** **(100.00)**

^a^ including only eyes that improved to VA ≥ 20/32 after subjective refraction

^b^ Retinal abnormalities such as: age-related macular degeneration, diabetic retinopathy, retinal detachment, macular hole, and chorioretinitis

Cataracts (39.1%) and refractive errors (29.1%) were the most frequent causes of visual impairment and blindness in this population. When analyzing each age group separately, we found that the main causes among those aged 45 years and more were cataracts (54.5%) while refractive errors were the main cause in adults aged 18 to 45 years (50.0%) and children up to 18 years old (37.1%).

The analysis of spherical equivalent from the 181 eyes classified as visually impaired or blind due to uncorrected refractive errors showed a mean of -0.58 ± 1.48 (median: -0.56) spherical diopters, ranging from -5.87 to + 2.50.

Regardless of visual status, cataract in either eye was detected in 213 individuals (10.1%), most of them aged 45 years old or more (97.2%). The overall frequency of cataract in the older group was 45.4% (207 persons).

Pterygium in either eye was detected in 226 individuals (10.8%) with important difference in the frequency of involvement in different age groups. Individuals aged between 18 and 45 years old had pterygium frequency of 13.0% (106 persons). Individuals aged 45 years or older had a pterygium frequency of 26.3% (120 persons). No case of pterygium was found in children up to 18 years of age.

## Discussion

The current study analyzed data collected during an eye care assistance project to indigenous communities living in remote areas of Brazil. It mapped an ocular epidemiological profile of the inhabitants of the Xingu Indigenous Park, including a robust number of participants. No previous study has described the ocular profile of indigenous population in such detail or have covered individuals from all age ranges.

Despite covering a relevant part of the Brazilian population living in the Xingu National Park, some limitations might be pointed. No data regarding sex of participants were provided restricting our analysis. No probabilistic sampling scheme on individual’s selection was performed which would improve the estimates accuracy. Studies with spontaneous demand sampling tend to overestimate the frequencies of diseases since the individuals with complaints are more prone to seek for evaluation. In this study, however, despite having spontaneous demand, we observed high coverage rates in the group aged from 18 to 44 years old group (75.6%) and in the group aged 45 years or more (93.3%). The high coverage rates increase the reliability of the results presented for those age groups.

The low response rate of individuals up to 18 years old (39.3%) is possibly a result of a cultural assumption that ocular disorders are exclusively related to age and do not affect children, as described elsewhere [17]. Despite the efforts to promote awareness about the importance of ocular exams in childhood, most parents chose to not bring children to the evaluation and this group was still little represented in the study.

The association between age and visual impairment and blindness found in the Xingu Indigenous population are in accordance with previous studies, with rates increasing with aging [1, 18–20]. The magnitude of blindness, however, indicate a worse ocular health in the indigenous population when compared to the overall worldwide population.

According to the most recent reports, the worldwide prevalence of blindness in childhood varies from 0.03 to 0.11%; in adults aged 18 to 44 years old varies from 0.12 to 0.32%; and in adults over 45 years old varies from 1.51 to 2.03% [1]. In the evaluated indigenous population, the burden of blindness was significantly higher than the worldwide statistics. The most recent national survey in the Brazilian Amazon Region showed a prevalence of blindness in adults 45 years and older of 2.8%, still significantly lower than the results from the current study [20]. As a result, public health policies designed aiming the decrease of blindness rates in general populations may not be efficient to cover minority populations as the indigenous, once specific data of those populations are often ignored when general estimates are calculated.

Following the results from national population-based studies and worldwide trends, cataract and uncorrected refractive errors were the main causes of visual impairment and blindness in the population [2, 18–20]. Main causes of blindness varied according to the age group with uncorrected refractive error showing a higher impact on younger individuals and cataracts on older individuals. Previous studies with Brazilian indigenous populations found prevalence of trachoma ranging from 28.0 to 56.4%, however, the only study performed at the Xingu Indigenous Park showed no trachoma cases, as the current study [13, 21–23].

Pterygium was a condition of interest as it is a disease highly associated with ultraviolet (UV) exposure [24]. Other studies carried out in Brazil reported prevalence of pterygium in adults aged 45 years and more of 11% in the Botucatu city located at latitude 22° S and 59% in the Parintins city located at 2° S [25, 26]. Taking into account the geographical position and the association between latitude and UV exposure, at the Xingu Indigenous Reserve located at latitude 12° S, an intermediate frequency of pterygium was observed (26.3%). Yet, the cumulative UV exposure increases with age and therefore higher frequencies of pterygium are expected in older individuals, which goes in accordance to our finding of no cases among children up to 18 years of age, low frequency among those aged 18 and 44 years old, and a higher frequency on those 45 years or older [27]. Moreover, a population profile is also a determinant for the pterygium development so that people who have outdoor occupations as most of the adults included in the current study tends to be more likely to develop the disease as the direct UV exposure is increased [26].

The Brazilian National Insurance program (SUS) counts with 34 local health-care units, called Indigenous Special Health Districts (DSEI) throughout the Indigenous territories, which provide primary care with multidisciplinary health teams including Indigenous community health workers, nurses, doctors, and dentists [28]. Our data highlights the importance of including visual screening into the regular practice of DSEIs.

## Conclusion

In conclusion, our study shows a higher frequency of visual impairment and blindness in the indigenous population when compared to worldwide estimates with most of the causes preventable and/or treatable. Therefore, blindness prevention programs should focus on accessibility to eye exam, cataract surgeries and eyeglass distribution for those groups. In this context, the Olhos do Xingu Project represents not only an intervention model in order to delivery eye care to remote regions of Brazil, but also an opportunity to study the ocular epidemiological profile of the country's indigenous populations.

## Data Availability

The data that support the findings of this study are available from Associação Médicos da Floresta—Brazil but restrictions apply to the availability of these data, which were used under license for the current study, and so are not publicly available. Data are however available from the authors upon reasonable request and with permission of Associação Médicos da Floresta—Brazil.

## Abbreviations

BCVA: Best-corrected visual acuity
DSEI: Indigenous Special Health Districts
MSVI: Moderate to severe visual impairment
SUS: Brazilian National Insurance
UV: Ultraviolet
UVA: Uncorrected visual acuity
VA: Visual acuity
